# Factors associated with subclinical inflammation of wrist joints in rheumatoid arthritis patients with low or no disease activity- a RA ultrasound registry study

**DOI:** 10.1186/s12891-023-06521-8

**Published:** 2023-05-30

**Authors:** Yu-Wei Wang, Jia-Feng Chen, Chi-Hua Ko, Tien-Tsai Cheng, Wen-Chan Chiu, Shan-Fu Yu, Chung-Yuan Hsu, Ying-Chou Chen

**Affiliations:** Department of Rheumatology, Kaohsiung Chang Gung Memorial Hospital, Chang Gung University College of Medicine, 123 Ta-Pei Road, Niao-Sung Dist. Kaohsiung 833, Kaohsiu, Taiwan

**Keywords:** Subclinical inflammation, Rheumatoid arthritis, Ultrasound, DAS 28, Disease activity

## Abstract

**Background:**

To evaluate the factors to predict subclinical inflammation of wrist joints in patients with RA who are in clinical remission or low disease activity.

**Methods:**

Gray scale and power Doppler ultrasound were performed on the dorsal radio-lunate of both wrists. The presence of synovitis, comorbidities, and use of disease modifying anti-rheumatic drugs were recorded. A Multivariable forward logistical regression model was used to identify factors associated with subclinical inflammation.

**Results:**

There were 1248 patients (1010 females, 238 males; mean age: 60.0 ± 10.5 years ). 57.4% of patients in complete remission and low disease activity had sonographic inflammation. Multivariable forward logistic regression analysis indicated that male sex, smoking are positively associated with inflammation and that age, alcohol consumption, and use of methotrexate, glucocorticoid, or a biological therapy are negatively associated with inflammation. Use of biological agents decreased the risk of inflammation by 40.9%.

**Conclusions:**

There was evidence of subclinical inflammation in most patients who were in low or no disease activity, those with biological therapy had lower risk of subclinical inflammation.

## Introduction

Approximately half of rheumatoid arthritis (RA) patients in clinical remission had disease flare in 24 months [1]. RA patients with flare generally have more radiographic progression than those in sustained remission. Thus, when an RA patient achieves remission, the clinician must know how to maintain remission to ensure the best possible outcome. Subclinical synovitis often resulted in clinical synovitis [2]. Previous studies with power Doppler ultrasound (PDUS) demonstrated active synovial inflammation in RA patients who were in clinical remission [3, 4]. Therefore, PDUS can be used to accurately predict subsequent radiographic progression [5]. Nonetheless, subclinical inflammation may continue when patients appear to be in clinical remission, and subsequent disease flare can occur.

There is concern about disease remission can accurately reflect disease state. Remission is characterized as the lack of synovial hyperemia and a lack of radiographic deterioration [6]. Ultrasound can detect synovitis in those without clinical synovitis [7]. Previous reported that a significant proportion of patients thought to be in clinical remission actually exhibited different grades of synovitis by ultrasound or magnetic resonance imaging (MRI), and some patients suffered from flares and joint damage during follow-up [3–5].

The present study aimed to identify factors affecting subclinical inflammation of wrist in patients with RA who are in clinical remission or low disease activity.

## Methods

This was a registry study of RA. The interim analysis of was conducted at CGMHK. The reporting of this study conforms to the STROBE statement [8]. This study was cross-sectional study approved by the local Institutional Review Board of Chang Gung Memorial Hospital in Kaohsiung, Taiwan. IRB No: 202000320B0. The Declaration of Helsinki was followed for all participants. Recruitment of the participants took place from December 1, 2020 to December 31, 2021., and the trial ended on December 31, 2021. Participants were requested to sign a written informed consent form approved by the institution ethics committee of Kaohsiung Chang Gang Memorial Hospital. The CONSORT guidelines were followed [9] and the CONSORT diagram was used to describe the flow of participants at each stage of the trial.

All patients underwent clinical examinations, laboratory assessments, and assessments of joints by the disease activity score 28 (DAS28). The use of medications and biological therapies, including etanercept, adalimumab, golimumab, tocilizumab, rituximab, and abatacept were recorded. Inclusion criteria were (1) those 1987 ACR criteria for rheumatoid arthritis [10] (2) those with DAS < 2.6 (remission) or 2.6 ≦ DAS＜3.2 (low disease activity ) [11]. Exclusion criteria were those with associated with infection or malignancy.

We choose bilaterally wrist joints modified from Peluso et al. at the radiocarpal joint [12]. Gray scale (GS) synovitis was scored *via* US scans in random order by an experienced observer (JFC) who was blinded to the clinical data. The GS synovitis and PDUS was graded as 0–3 by the system of Szkudlarek and colleagues [13]. We use (GSUS + PDUS) ≧ 2 based on the report by Hilde Berner Hammer [14].

and we defined ≧ 2as subclinical inflammation [15].

### Statistical analyses

SPSS software (version 20.0; SPSS, Inc., Chicago, IL) was used to perform all statistical analyses. Normally distributed data are presented as means ± standard deviations. The *t*-test was used for statistical comparisons. The chi-square test for categorical variables. Forward logistic regression was used to adjust for variables predictive of sonographic inflammation.

Intra-observer reliability was evaluated with a two-way mixed effects model using a definition for consistency that excluded the between-measure variance from the denominator variance and both single measures. Average intra-class correlation coefficients (ICCs) were calculated for GSUS and PDUS synovitis scores. The weighted κ values were calculated on a joint-by-joint basis for PDUS scores. The ICC and κ values were compared; scores greater than 0.60 were considered good and scores greater than 0.8 as very good.

## Results

We enrolled 1248 patients (85.6% females) who had rheumatoid arthritis (Table 1). The mean age was 60.0 ± 10.5 years and the mean BMI was 23.27 ± 3.72. There were 202 patients (16.2%) in complete remission and 214 patients (17.1%) had low disease activity (2.6 < DAS28 ≤ 3.2). Most patients (67.5%) were using methotrexate (MTX) as a disease-modifying, anti-rheumatic drug (DMARD).

**Table 1:** Baseline demographic and clinical characteristics of patients with RA

Characteristic	N = 416
**Age (years), mean ± SD**	60.49 ± 11.45
**Body mass index (kg/m2), mean ± SD**	23.58 ± 3.71
**Rheumatoid arthritis follow-up, mean ± SD**	9.42 ± 4.65
**Sex (female %)**	336(80.8)
**Smoking (n, %)**	28(6.7)
**Alcohol consumption (n, %)**	28(6.7)
**Diabetes (n, %)**	22(5.3)
**Hypertension (n, %)**	104(25)
**Cardiovascular disease (n, %)**	18(4.3)
**Pulmonary disease (n, %)**	10(2.4)
**Liver disease (n, %)**	14(3.4)
**Gastrointestinal disease (n, %)**	15(3.6)
**DAS28, mean ± SD**	2.47 ± 0.56
**Complete remission (DAS28** $\leqq$ **2.6)**	202
**Low disease activity(2.6 < DAS28** $\leqq$ **3.2)**	214
**Use of other RA mediations**	
**Methotrexate (n, %)**	278(66.8)
**Hydroxychloroquine (n, %)**	170(40.9)
**Leflunomide (n, %)**	48(11.5)
**Cyclosporin (n, %)**	8(1.9)
**Sulfasalazine (n, %)**	10(2.4)
**Glucocorticoid (n, %)**	336(80.8)
**Biological therapy (n, %)**	111(26.7)

**Fig. 1:**
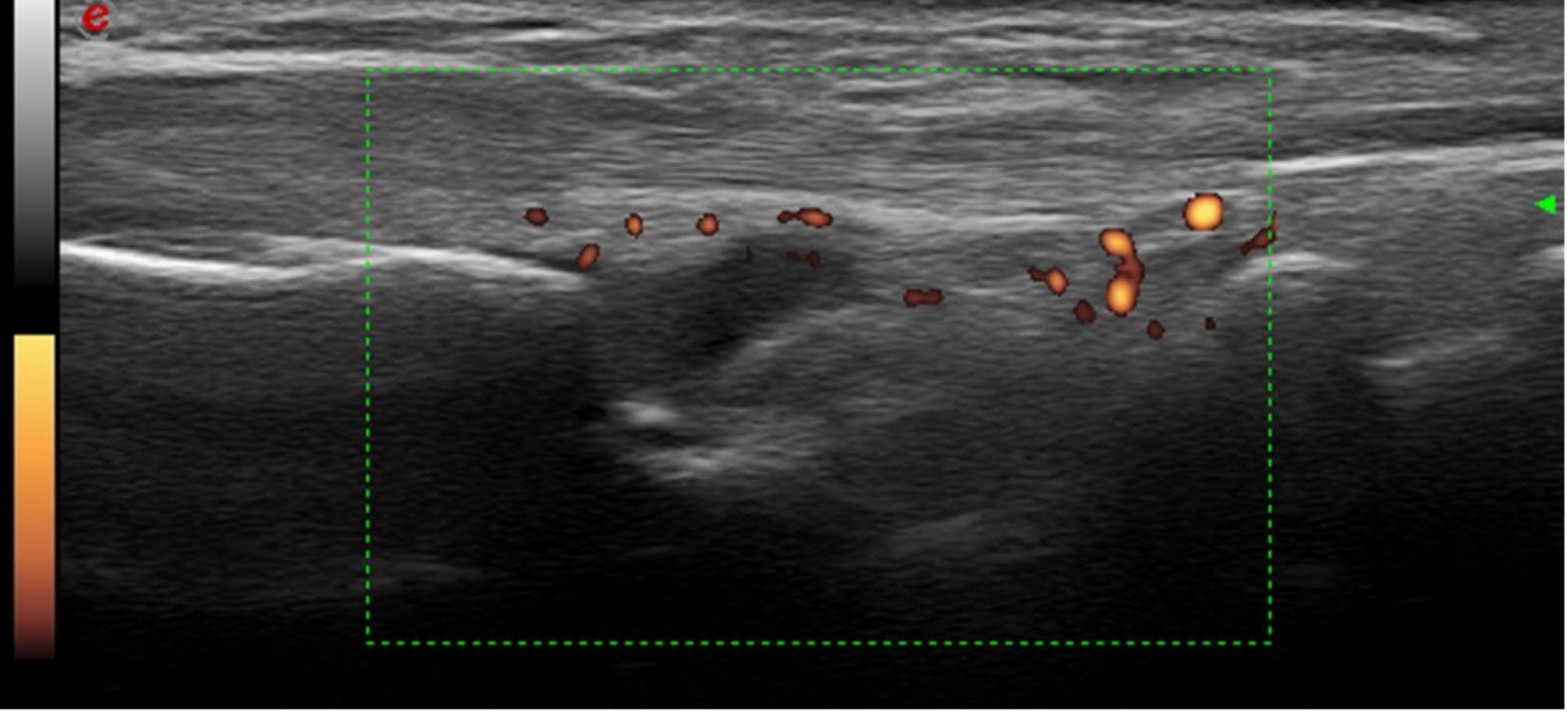
Ultrasound in a 40 -year-old woman with RA. The DAS28 is 2.6, while synovial hypertrophy is grade 2, power Doppler is grade 2

US was performed on radio-lunate joint of the wrist, interestingly, 57.4% of the patients in complete remission and lower disease activity had subclinical inflammation (Fig. 1). We used multivariable forward logistic regression to identify factors associated with sonographic inflammation (Table 2). The results indicate that male sex (OR: 1.69, 95% confidence interval [CI]: 1.37–2.09, *p* = 0.001), smoking (OR: 1.69, 95% CI: 1.21–2.35, *p* = 0.002) were positively associated with subclinical inflammation. In contrast, advanced age (OR: 0.98, 95% CI: 0.98–0.99, *p* = 0.001), alcohol consumption (OR: 0.55, 95% CI: 0.35–0.84, *p* = 0.006), use of MTX (OR: 0.83, 95% CI: 0.71–0.97, *p* = 0.020), use of a glucocorticoid (OR: 0.60, 95% CI: 0.52–0.69, *p* = 0.001), and use of a biological therapy (OR: 0.59, 95% CI: 0.50–0.68, *p* = 0.001) were negatively associated with inflammation.

**Table 2:** Multivariable forward logistic regression analysis of factors associated with subclinical inflammation

Variable	Regression coefficient	Standard error	Wald	*p* value	Odd ratio (95% CI)
Sex	0.52	0.10	23.93	0.001	1.69(1.37–2.09)
Age	-0.01	0.00	13.48	0.001	0.98 (0.98–0.99)
Body mass index (kg/m^2^)	-0.01	0.00	0.56	0.451	0.99 (0.97–1.01)
Smoking	0.52	0.16	9.69	0.002	1.69 (1.21–2.35)
Alcohol consumption	-0.59	0.21	7.44	0.006	0.55(0.35–0.84)
Methotrexate	-0.18	0.07	5.45	0.020	0.83(0.71–0.97)
Hydroxyxhloroquine	0.07	0.07	0.94	0.332	1.07 (0.93–1.23)
Sulphasalazine	-0.28	0.16	2.86	0.091	0.75 (0.54–1.04)
Leflunomide	0.05	0.10	0.24	0.622	1.05 (0.42–1.100)
Cyclosporine	-0.64	0.46	1.88	0.170	0.52 (0.21–1.31)
Glucocorticoid use	-0.50	0.07	48.27	0.001	0.60(0.52–0.69)
Biological therapy	-0.52	0.07	48.83	0.001	0.59 (0.50–0.68)

## Discussion

Several studies have shown that GSUS and PDUS assessment of RA patients is useful for assessment of inflammation [16–18]. In particular, PDUS and/or GSUS signals can be used to assess hand joints in patients with RA [19]. Previous publications validated the use of global sonographic joint counts for synovitis [20–22]. However, there is currently no universally accepted combination of joints to be included in a global US score for synovitis [23]. In this study, we focused on the wrist joints, rather than the larger joints.

There had reports that more than 40% of patients with RA who are in clinical remission have elevated PDUS signals, and this explains why a proportion of these patients develop radiographic progression during follow-up [3, 4]. PDUS can provide additional useful information in clinical examination of RA patients by allowing an earlier diagnosis and establishing when a patient is in true remission. Synovial hypertrophy (SH), scored by GSUS, seems less specific, and healthy individuals are frequently given grades of 1 [24]. At present, there is no clear criteria of active synovitis based on US results, but from expert’s views, they suggested the sum of the SH and PDUS signal can be used to define active synovitis [25].

In our study, over half of the RA patients had signs of active synovitis, even those with low disease activity and those in complete remission. Male patients also tended to have higher disease activity than females, although 85.6% of our patients were females. This finding is consistent with a study by Tokunaga et al. [26], which showed that male RA patients experienced no improvements in the modified health assessment questionnaire after treatment. We also found that old age was associated with less subclinical inflammation, which is different from previous reports [27, 28]. The reasons may be more glucocorticoid use in these group. Our results also indicated that smoking was associated with increased subclinical inflammation, consistent with another clinical study which showed that smoking was associated with not being in remission [29]. Alcohol consumption was associated with less subclinical inflammation, consistent with a previous clinical study which showed that alcohol may be protective against various kinds of arthritis [30].

Methotrexate is known to decrease RA activity, [31] as also shown by the subclinical inflammation of the current study. We also found that glucocorticoid therapy was associated with lower subclinical inflammation. In agreement, a previous clinical study showed that starting treatment with high-dose glucocorticoids leads to a better clinical response within 3–6 months than starting with a lower dosage [32]. Importantly, we found that use of biological DMARDs can decrease subclinical inflammation risk by 40.9% (OR: 0.591). Therefore, combined clinical and US assessments appear effective in identification of individuals in remission who may be suitable for reduced doses of DMARDs, which may be associated with adverse effects. In other words, US could provide a safe method of monitoring RA patients for subclinical disease flare-ups [33]. So we hypothesized that a combination of PDUS and DAS28 evaluations for targeted therapy is recommended to avoid future disease flare-ups and joint destruction.

The study had some limitations. Given the cross-sectional design of this study, heterogeneous study population, and the incomplete information on treatment (i.e. no data on the treatment for comorbidity, no dose or duration), it is almost impossible to speculate the causality or underlying mechanism of the significant associations between subclinical inflammation and background variables identified by logistic regression. No anti-CCP or HAQ were recorded. The inclusion of patients with different treatment regimens is an important confounding factor. One third of the cohort was under treatment with biologics and nearly 16% of included patients has DAS based remission. So the analysis should be conducted after stratification based in the therapy scheme.

In conclusion, our study confirms the value of US for detecting the subclinical disease activity, which may persist even after years of apparent clinical remission. US may be particularly helpful for identifying joints that have greater damage, and use of biological DMARDs can decrease subclinical inflammation risk.

## Data Availability

All data generated or analyzed during this study are presented in the manuscript. Please contact the corresponding author for access to data presented in this study.

## Abbreviations

DAS28: disease activity score in 28 joints
RA: rheumatoid arthritis
GS: Gray scale
PDUS: power Doppler ultrasound
MRI: magnetic resonance imaging
ACR: American college of Rheumatology
MTX: methotrexate
DMARD: disease-modifying, anti-rheumatic drug
OR: odds ratio
CI: confidence interval
SH: Synovial hypertrophy
anti-CCP: anti-cyclic citrullinated peptide antibody
HAQ: Health Assessment Questionnaire
US: ultrasound

## References

[1] Molenaar ET, Voskuyl AE, Dinant HJ, Bezemer PD, Boers M, Dijkmans BA (2004). Progression of radiologic damage in patients with rheumatoid arthritis in clinical remission. Arthritis Rheum.

[2] Rogier C, Wouters F, van Boheemen L, van Schaardenburg D, de Jong PHP, van der Helm-van Mil AHM (2021). Subclinical synovitis in arthralgia: how often does it result in clinical arthritis? Reflecting on starting points for disease-modifying anti-rheumatic drug treatment. Rheumatology (Oxford).

[3] Saleem B, Brown AK, Keen H, Nizam S, Freeston J, Karim Z (2009). Disease remission state in patients treated with the combination of tumor necrosis factor blockade and methotrexate or with disease-modifying antirheumatic drugs: a clinical and imaging comparative study. Arthritis Rheum.

[4] Brown AK, Quinn MA, Karim Z, Conaghan PG, Peterfy CG, Hensor E (2006). Presence of significant synovitis in rheumatoid arthritis patients with disease-modifying antirheumatic drug-induced clinical remission: evidence from an imaging study may explain structural progression. Arthritis Rheum.

[5] Brown AK, Conaghan PG, Karim Z, Quinn MA, Ikeda K, Peterfy CG (2008). An explanation for the apparent dissociation between clinical remission and continued structural deterioration in rheumatoid arthritis. Arthritis Rheum.

[6] Haavardsholm EA, Lie E, Lillegraven S (2012). Should modern imaging be part of remission criteria in rheumatoid arthritis?. Best Pract Res Clin Rheumatol.

[7] Di Matteo A, Corradini D, Mankia K. What is the value of Ultrasound in individuals ‘At-Risk’ of Rheumatoid Arthritis who do not have clinical Synovitis? Healthcare (Basel). 2021;9(6). 10.3390/healthcare9060752 PMC8233794 34207207

[8] von Elm E, Altman DG, Egger M, Pocock SJ, Gotzsche PC, Vandenbroucke JP (2007). The strengthening the reporting of Observational Studies in Epidemiology (STROBE) statement: guidelines for reporting observational studies. PLoS Med.

[9] Boutron I, Altman DG, Moher D, Schulz KF, Ravaud P, Group CN (2017). CONSORT Statement for Randomized trials of nonpharmacologic treatments: a 2017 update and a CONSORT extension for nonpharmacologic trial abstracts. Ann Intern Med.

[10] Arnett FC, Edworthy SM, Bloch DA, McShane DJ, Fries JF, Cooper NS (1988). The american Rheumatism Association 1987 revised criteria for the classification of rheumatoid arthritis. Arthritis Rheum.

[11] Anderson J, Caplan L, Yazdany J, Robbins ML, Neogi T, Michaud K (2012). Rheumatoid arthritis disease activity measures: American College of Rheumatology recommendations for use in clinical practice. Arthritis Care Res (Hoboken).

[12] Peluso G, Michelutti A, Bosello S, Gremese E, Tolusso B, Ferraccioli G (2011). Clinical and ultrasonographic remission determines different chances of relapse in early and long standing rheumatoid arthritis. Ann Rheum Dis.

[13] Szkudlarek M, Narvestad E, Klarlund M, Court-Payen M, Thomsen HS, Ostergaard M (2004). Ultrasonography of the metatarsophalangeal joints in rheumatoid arthritis: comparison with magnetic resonance imaging, conventional radiography, and clinical examination. Arthritis Rheum.

[14] Hammer HB, Kvien TK, Terslev L (2017). Ultrasound of the hand is sufficient to detect subclinical inflammation in rheumatoid arthritis remission: a post hoc longitudinal study. Arthritis Res Ther.

[15] Ko CH, Chen JF, Cheng TT, Lai HM, Chen YC (2018). Biological tapering and sonographic flare in rheumatoid arthritis. J Investig Med.

[16] Saleem B, Brown AK, Keen H, Nizam S, Freeston J, Wakefield R (2011). Should imaging be a component of rheumatoid arthritis remission criteria? A comparison between traditional and modified composite remission scores and imaging assessments. Ann Rheum Dis.

[17] Wakefield RJ, Freeston JE, Hensor EM, Bryer D, Quinn MA, Emery P (2007). Delay in imaging versus clinical response: a rationale for prolonged treatment with anti-tumor necrosis factor medication in early rheumatoid arthritis. Arthritis Rheum.

[18] Filippucci E, Cipolletta E, Mashadi Mirza R, Carotti M, Giovagnoni A, Salaffi F (2019). Ultrasound imaging in rheumatoid arthritis. Radiol Med.

[19] Felson D (2012). Defining remission in rheumatoid arthritis. Ann Rheum Dis.

[20] Naredo E, Collado P, Cruz A, Palop MJ, Cabero F, Richi P (2007). Longitudinal power Doppler ultrasonographic assessment of joint inflammatory activity in early rheumatoid arthritis: predictive value in disease activity and radiologic progression. Arthritis Rheum.

[21] Gutierrez M, Filippucci E, Salaffi F, Di Geso L, Grassi W (2011). Differential diagnosis between rheumatoid arthritis and psoriatic arthritis: the value of ultrasound findings at metacarpophalangeal joints level. Ann Rheum Dis.

[22] Naredo E, Bonilla G, Gamero F, Uson J, Carmona L, Laffon A (2005). Assessment of inflammatory activity in rheumatoid arthritis: a comparative study of clinical evaluation with grey scale and power doppler ultrasonography. Ann Rheum Dis.

[23] Mandl P, Naredo E, Wakefield RJ, Conaghan PG, D’Agostino MA (2011). A systematic literature review analysis of ultrasound joint count and scoring systems to assess synovitis in rheumatoid arthritis according to the OMERACT filter. J Rheumatol.

[24] Ten Cate DF, Luime JJ, Swen N, Gerards AH, De Jager MH, Basoski NM (2013). Role of ultrasonography in diagnosing early rheumatoid arthritis and remission of rheumatoid arthritis–a systematic review of the literature. Arthritis Res Ther.

[25] Dougados M, Devauchelle-Pensec V, Ferlet JF, Jousse-Joulin S, D’Agostino MA, Backhaus M (2013). The ability of synovitis to predict structural damage in rheumatoid arthritis: a comparative study between clinical examination and ultrasound. Ann Rheum Dis.

[26] Tokunaga T, Miwa Y, Nishimi A, Nishimi S, Saito M, Oguro N (2015). Sex differences in the Effects of a Biological Drug for Rheumatoid Arthritis on Depressive State. Open Rheumatol J.

[27] Radovits BJ, Fransen J, van Riel PL, Laan RF (2008). Influence of age and gender on the 28-joint disease activity score (DAS28) in rheumatoid arthritis. Ann Rheum Dis.

[28] Radovits BJ, Kievit W, Fransen J, van de Laar MA, Jansen TL, van Riel PL (2009). Influence of age on the outcome of antitumour necrosis factor alpha therapy in rheumatoid arthritis. Ann Rheum Dis.

[29] Inoue Y, Nakajima A, Tanaka E, Inoue E, Kobayashi A, Hoshi D (2015). Effect of smoking on remission proportions differs between male and female patients with rheumatoid arthritis: a study based on the IORRA Survey. J Rheumatol.

[30] Huidekoper AL, van der Woude D, Knevel R, van der Helm-van Mil AH, Cannegieter SC, Rosendaal FR (2013). Patients with early arthritis consume less alcohol than controls, regardless of the type of arthritis. Rheumatology (Oxford).

[31] Oh K, Ito S, Unno M, Kobayashi D, Azuma C, Abe A (2015). The rate of decrease in the disease activity of rheumatoid arthritis during treatment with adalimumab depends on the dose of methotrexate. Intern Med.

[32] Albrecht K, Callhoff J, Schneider M, Zink A (2015). High variability in glucocorticoid starting doses in patients with rheumatoid arthritis: observational data from an early arthritis cohort. Rheumatol Int.

[33] Marks JL, Holroyd CR, Dimitrov BD, Armstrong RD, Calogeras A, Cooper C (2015). Does combined clinical and ultrasound assessment allow selection of individuals with rheumatoid arthritis for sustained reduction of anti-tumor necrosis factor therapy?. Arthritis Care Res (Hoboken).

